# Supplementary material for: Manipulating exchange bias in 2D magnetic heterojunction for high-performance robust memory applications
Source: Nat Commun. 2023 Apr 17;14:2190. doi: 10.1038/s41467-023-37918-7 (PMC10110563; doi:10.1038/s41467-023-37918-7)
Supplement: Supplementary file 1 — Supplementary information [file 41467_2023_37918_MOESM1_ESM.pdf]

Supplementary information for

Manipulating Exchange Bias in 2D Magnetic Heterojunction for High-performance Robust Memory Applications

Supplementary Note

Section 1. Characterization of bulk crystals

Section 2. Characterizations of heterostructures

Section 3. Parametrization of the force fields for FGT and FPSe intra and inter flake interatomic interactions.

Section 4. Molecular Dynamics simulations

Section 5. Conversion of laser power and pressure

Supplementary Figures

Fig. 1: Elemental analysis of FPSe bulk crystals.

Fig. 2: Elemental analysis of FGT bulk crystals.

Fig. 3: Magnetization measurements of FPSe and FGT bulk crystals.

Fig. 4: The Raman spectroscopy for FGT (green), FPSe (blue), and FPSe/FGT heterostructure (red).

Fig. 5: The unit cell for FPSe and for FGT built using BURAI (Quantum Expresso GUI).

Fig. 6: Layers in Molecular Dynamics simulations using LAMMPS for FPSe (bottom) and for FGT (top).

Fig. 7: Strain and interlayer distance evolution in the laser shock MD simulation.

Fig. 8: Characterization of FGT heterostructure after LS.

Fig. 9: Characterization of FPSe and FPSe/FGT heterostructure after LS.

Fig. 10: The optical microscope image, the atomic force microscope image, and the

Raman spectroscopy characterization of sample A.

Fig. 11: The Kerr hysteresis loops of pure FGT.

Fig. 12: The schematic diagram of the M-B model.

Fig. 13: The thickness dependences of H_{EB} in AFM/FM systems.

Fig. 14: The optical microscope image and the atomic force microscope image of sample B.

Fig. 15: The evolution of the coercive field on temperature and pressure before and after LS.

Fig. 16: The optical microscope image and the atomic force microscope image of FPSe/FGT/h-BN/FGT heterostructure (pristine device and LS modulation device).

Fig. 17: The optical microscope image and the atomic force microscope image of FPSe/FGT/h-BN/FGT heterostructure (thickness modulation device).

Fig. 18: TMR curves measurement of FPSe/FGT/h-BN/FGT at different temperatures before and after LS.

Fig. 19: TMR measurement of thickness modulation device.

Fig. 20: TMR curves measurement of FGT/h-BN/FGT at 5 K with a bias current of 3 μA.

Fig. 21: The optical microscope image, the atomic force microscope image, and MOKE signal for sample C.

Fig. 22: RMCD signal of FPSe/FGT heterostructure (sample D).

Fig. 23: RMCD signal of FPSe/FGT heterostructure (sample E)

Fig. 24: Schematic diagram of Magnetic Random Access Memory (MRAM).

Supplementary Table

Table 1: Atomic positions (Å) in simulation cells at the ground state (image from BURAI).

Table 2: Energy states for different atomic configurations.

Table 3: Interatomic interactions model parameters.

Table 4: The summary of the peak pressures at 4 different laser intensities.

Table 5: The performance comparison before and after laser shocking.

Section S1. Characterization of bulk crystals

As shown in Supplementary Fig. 1 and 2, the quality, orientation, topography, and composition of the FGT and FPSe bulk crystals were checked by the SEM equipped with an EDS. The magnetizations of FGT and FPSe were measured by a superconducting quantum interference device (SQUID) from 4.2 K to 300 K with the magnetic field sweeping up to 9 T. Magnetization measurements of FPSe and FGT bulk crystals are shown in Supplementary Fig. 3. The T_N of FPSe and the T_C of FGT were extracted as 110 K and 230 K, respectively.

Section S2. Characterizations of heterostructures

The morphology and thickness of the three samples (sample A, sample B, and sample C) mentioned in the main text were characterized by atomic force microscopy.

As is shown in Supplementary Fig. 9, the thicknesses of FGT and FPSe are 18 nm and 24.4 nm in sample A, respectively. Sample A demonstrated the enhancement of pressure-induced magnetism coupling using out-of-plane pressure via laser shocking. The enhanced interlayer coupling of the FPSe/FGT heterostructure led to an impressive improvement of the EB field and blocking temperature. A comparison for samples before and after laser shocking is summarized in Supplementary Table 5. Supplementary Fig. 10 shows the typical MOKE curves of FGT (thickness: 23.4 nm) as a function of the magnetic field (B) from 5 K to 200 K. And their Kerr hysteresis loops remained symmetric relative to the zero point along the B-axis, suggesting a lack of H_{EB}.

To investigate the non-localized coupling mechanism of the horizontal pinning, we fabricated FPSe/FGT heterostructures with isolated FGT (sample C), for comparison to the connected FGT (sample B) in terms of the Kerr signals. The optical

images and atomic force microscope images of sample B and C are shown in Supplementary Fig. 6 and 7, respectively. The MOKE signals for isolated FGT, connected FGT, and FPSe/FGT heterostructures of sample C were measured at 5 K. When FGT is connected to FPSe/FGT heterostructures, a consistent EB effect occurs. In contrast, FGT is not affected by the magnetic properties of the heterostructure. These results show that as long as the FGT is connected to FPSe/FGT, the extent of transverse propagation can be considerable, even exceeding 100 μm. In contrast, when there are cracks between FGT and FPSe/FGT heterostructures, the EB effect is absent in the isolated FGT, indicating that the connection is a necessary condition for the transverse propagation.

Section S3. Parametrization of the force fields for FGT and FPSe intra- and inter-flake interactions of FGT/FPSe.

The FPSe/FGT system has no dedicated force fields models in the literature. Therefore, we have built up our simplified force fields model that consists of 2-(Lennard-Jones) and 3-body (Axilrod Teller) interactions to represent the intraflake interatomic interactions and Lennard-Jones interlayer interactions. The modeling of the employed force field was divided into three main steps. The first step is determining the ground state of the atomic system via quantum mechanics self-consistency calculations. The second step consists of determining the energy of the same system in strained conditions also using quantum mechanics self-consistency calculations. The third step is to perform parameters search such that the energy differences between the different states of the atomic system determined in the first and second steps are close to the differences obtained employing the 2 (Lennard-Jones) and 3 -body (Axilrod Teller) interactions for the intralayer interatomic interactions, and Lennard-Jones

interflake interactions in Molecular Dynamics simulations.

S.3.1. First step

We initially assembled the unit cell structure for each 2D material (Supplementary Fig. 5), stacking one FGT unit cell stacked on top and close to a FPSe unit cell, and run cell relaxation simulations to obtain the ground state configuration for the single flakes alone and the stacked system.

For the FGT, we used 6 K-points for all spatial dimensions while for FPSe we used 7 K-points for all spatial dimensions in the real space. We performed the quantum mechanics self-consistent field (SCF) calculations employing a norm-conserving pseudopotential of the PBE exchange-correlation flavor for Te atoms; an ultrasoft (PBE) pseudopotential for Fe and P atoms; an ultrasoft (PBE) pseudopotential with 115.767 Ry charge cutoff and 19.619 Ry wavefunction cutoff for Ge atoms; an ultrasoft (PBE) pseudopotential with 117.426 Ry charge cutoff and 22.216 Ry wavefunction cutoff for Se atoms.

The unit cell employed for FGT was in a hexagonal Bravais lattice with A = 3.991 Å, and C = 18.931 Å. The unit cell employed for FPSe was a triclinic one with A = 6.166 Å, B = 10.680 Å, C = 12.856 Å, $\alpha = 90^{\circ}$, $\beta = 103.966^{\circ}$, and $\gamma = 90^{\circ}$. The atomic positions for the optimized configuration (Supplementary Table 1), can then be determined via self-consistent calculations.

S.3.2. Second step

We then executed the self- consistent calculations for 3 different atomic positions configurations with distinct cell dimensions, i.e., different strain levels. The energy calculation results for all these configurations are shown in Supplementary Table 2 for

FGT.

S.3.3. Third step

Employing identical cell geometry and atomic positions as in QM SCF calculations, we built the Molecular Dynamics (MD) simulations for each cell tested in QM. We assigned multiple combinations of parameters (ϵ_s and Z_s) using an array sampling in order to guarantee a good spread of parameters over the testing intervals. After all simulations were performed in MD, for all atomic configurations previously simulated in QM, we compared the states energy differences as done in Table S1, and the force field model parameters that resulted in the smallest errors between the MD and QM differences was selected as the final parameters (Supplementary Table 3).

The MD simulations (Supplementary Fig. 6) could then be performed after establishing the atomic system, and the interatomic interactions could be represented using the parameters determined with the above procedure.

Section S4. Molecular Dynamics simulations

The inter-layer coupling was studied employing Molecular Dynamics (MD) simulations. The simulations were designed in a 175 Å × 175 Å × 65 Å cell volume with periodic boundary conditions in all dimensions, and the time step was 0.3 fs. In this volume, a lower part (small *z* value) of FPSe had its lower Se atoms pinned for the z-direction. Then we intercalated two FGT and one FPSe on top of the pinned initial layer. Two- (Lennard-Jones, Eq. (1)) and three-body (Axilrod-Teller, Eq. (2)) interatomic interactions models were then set for each of the layers[1]. Dispersive Lennard Jones interactions were employed for interatomic interactions between neighbor layers[2]. An NVT ensemble (with a constant number of atoms, volume, and temperature) was employed.

$$E_{ij} = 4\varepsilon\left[\left(\frac{\sigma}{r_{ij}}\right)^{12} - \left(\frac{\sigma}{r_{ij}}\right)^{6}\right], r_{ij} < r_c \tag{1}$$

$$E_{ijk} = \frac{Z\left(1+3\cos\theta_i \cos\theta_j \cos\theta_k\right)}{\left(r_{ij}r_{ik}r_{jk}\right)^3} \tag{2}$$

Where the E_{ij} and E_{ijk} are two-body potentials and three-body dispersion potentials. the ε and σ parameters are characteristic of the strength of intermolecular interaction and molecular size, respectively. θ refers to the inside angles of a triangle formed by three molecules $i, j,$ and $k,$ and Z is the non-additive coefficient. r_{ij}, r_{ik}, and r_{jk} are the distance between the *i, j,* and *k* molecules. r_c is the cut-off radius of the interaction potential.

The coupling between flakes indicates that there are two possible equilibrium states. The existence of such equilibrium states is due to the nature of the inter-flake interatomic interactions nature. The relaxed/unstrained state presents both a weak repulsion force between the Se/Te flake edge atoms pairs which is counterbalanced by a weak attraction force between the P/Te, P/Fe, and Fe/Se atoms pairs. On the other hand, the strained equilibrium configuration presents a strong repulsion between the Se/Te atoms pairs that is counterbalanced by a strong attraction between the P/Te, P/Fe, and Fe/Se atoms pairs.

At the strained equilibrium state, the simultaneous attraction between the Fe atoms from the FGT and P atoms from the FPSe flakes, as well as repulsion between the Se atoms from the FPSe flakes and Te atoms from the flakes draw the Se and P atoms get closer in their z-axis coordinates, with respect to the unstrained configuration in the FPSe flake, and the Fe atoms closer to the Te atoms in their z-axis coordinates in the FGT flakes.

Subsequently, a simulation was conducted including a shock pulse of 14 GPa

(similar to the experimental condition) and the shock pressure lasted for 150 fs (500 time steps) over the top FGT flake, which was initially positioned 3 Å above the unstrained flake's equilibrium position to check if the shock can drive the equilibrium state transitions.

The system must overcome an energy barrier caused by the stronger inter-flake repulsion interaction between Se/Te atoms pairs until a stronger attraction term starts to be significant at a smaller inter-flake distance (~4.2 Å). The 14 GPa pulse shock can overcome the inter-states energy barrier, making the multi-flake FPSe/FGT system equilibrated at a larger strain state and lower inter-flake spacing.

Supplementary Fig. 7 shows the evolution of out-of-plane strains for each flake in the shock simulation as well as the interlayer distance over time. There is a slight compressive strain overshoot at around 10 ps. The bottom layer of FPSe did not present this overshoot because of the z-pinned applied boundary condition. Supplementary Fig. 7 also shows that the magnitude of out-of-plane strain magnitude increases as soon as the shocking occurs.

Section S5. Conversion of laser power and pressure

The acting time of laser-induced plasma shock wave on a metallic material is extremely short, which is within tens of nanoseconds in general. In order to calculate the pressure of laser-induced plasma shock wave during laser shock process, a numberof calculation models of shock wave pressure were proposed[3]. Among them, the one-dimensional model of shock wave pressure proposed by Fabbro [4] is well known and most universally acknowledged, which can reflect the change law of the peak pressure of laser-induced plasma shock waves with the laser pulse energy. The calculation formula for the peak pressure of laser-induced plasma shock waves is as

follows:

$$P_{\max}(GPa) = 0.01(\frac{\alpha}{2\alpha+3})^{1/2} z^{1/2}(g \cdot cm^{-2} \cdot s^{-1}) I^{1/2}(GW \cdot cm^{-2}) \tag{3}$$

where, $P_{\max}$ is the peak pressure of laser-induced plasma shock wave; α is the fraction of absorbed laser pulse energy (typically sets to 0.1), and I is the laser intensity. And z is the combined acoustic impedance of the metallic material and the confinement layer, which can be calculated by the following relation:

$$\frac{2}{z} = \frac{1}{z_1} + \frac{1}{z_2} \tag{4}$$

where $z_{1,2}$ represents target materials (graphite) and constraint layer (glass), respectively[5, 6].

The calculation formula of laser power irradiance can be expressed as follows:

$$I = \frac{4E}{\pi D^2 \tau} \tag{5}$$

where, I is the laser intensity, E is the laser pulse energy, D is the laser spot diameter, and τ is the pulse duration.

Here, we used 4 different laser intensities within the pulse duration τ = 10 ps and D = 300 μm. Laser pulse energe is 80 μJ (10 % loss) and pulse frequency is 125 kHz. Table S4 lists the peak pressures under 4 different laser intensities.

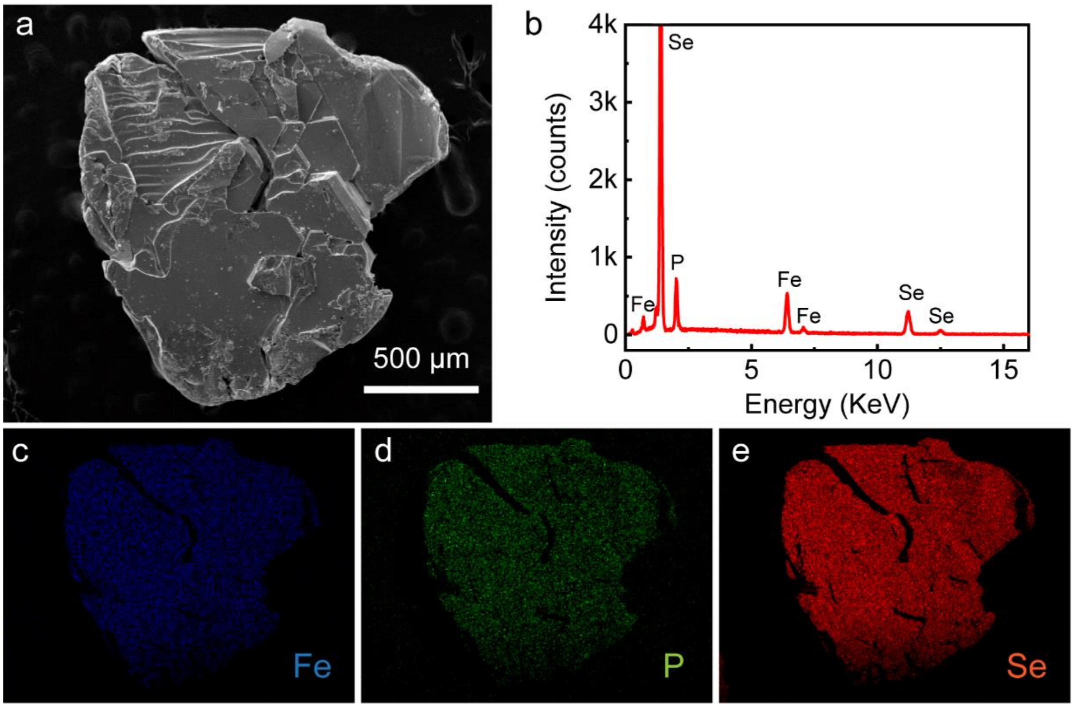

Supplementary Fig. 1 | Elemental analysis of FPSe bulk crystals. SEM image (a), EDS map (b) and EDS elemental analysis (c-e) of the FPSe bilk crystals respectively.

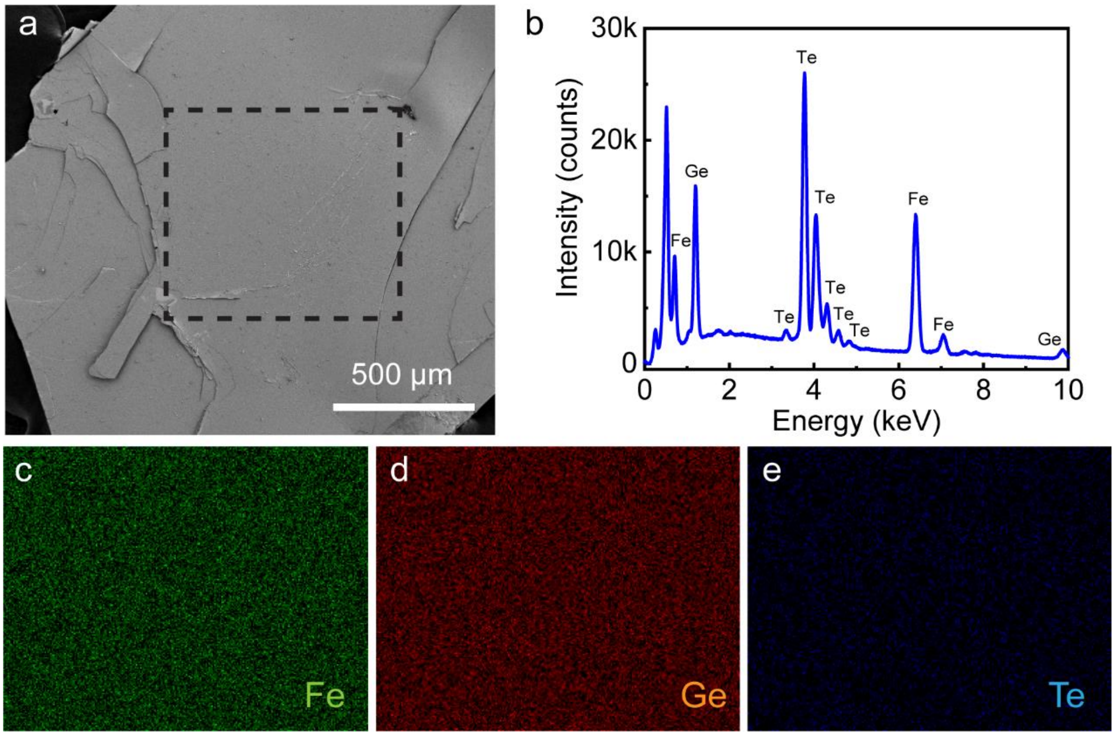

Supplementary Fig. 2 | Elemental analysis of FGT bulk crystals. SEM image (a), EDS map (b) and EDS elemental analysis (c-e) of the FGT bulk crystals respectively. The black box in (a) represent the test range of EDS elemental analysis (c-e).

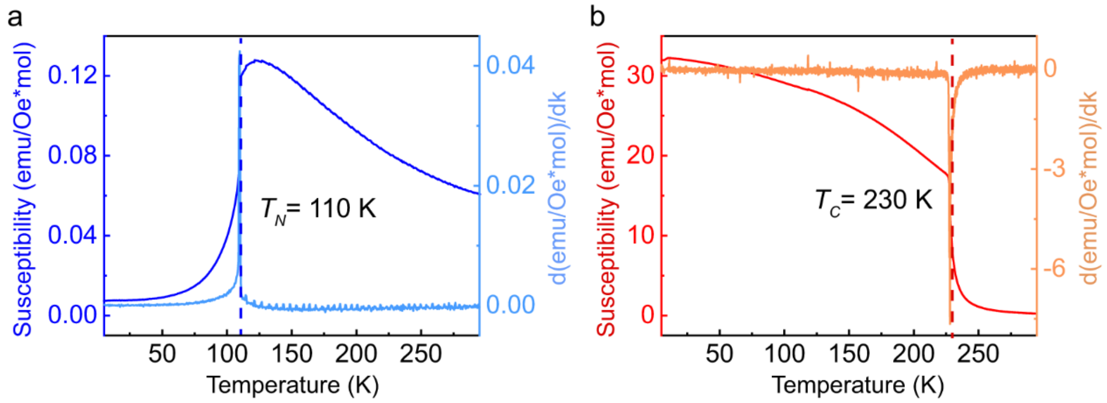

Supplementary Fig. 3 | Magnetization measurements of FPSe and FGT bulk crystals. a, b Magnetic susceptibility as a function of temperature for FGT (**a**) and FPSe (**b**) single crystals along the c-axis. The T_C of FGT and the T_N of FPSe were extracted as 230 K and 110 K, respectively.

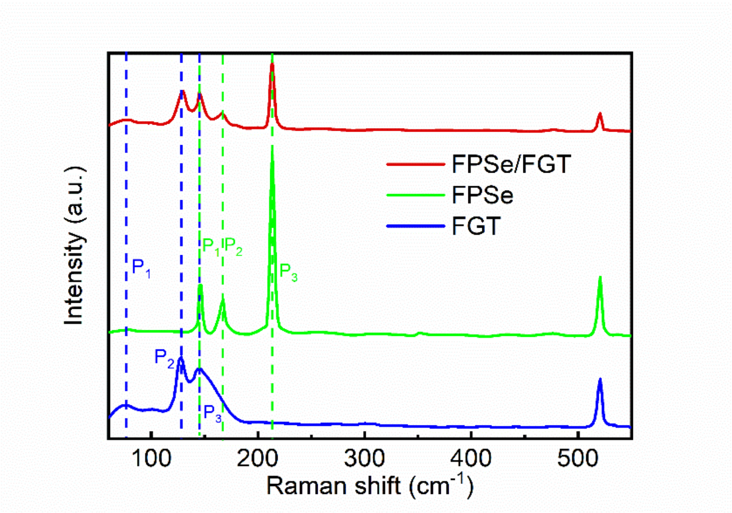

Supplementary Fig. 4 | Characterization of flakes. The Raman spectroscopy for FGT (green), FPSe (blue), and FPSe/FGT heterostructure (red). P_1, P_2, and P_3 marked in blue represent the characteristic peaks E_{2g}^1, E_{2g}^2, and A_{1g}^1 of FGT flake. P_1, P_2, and P_3 marked in green represent the characteristic peak E_g, A_{1g}, and A_{1g} of FPSe flake.

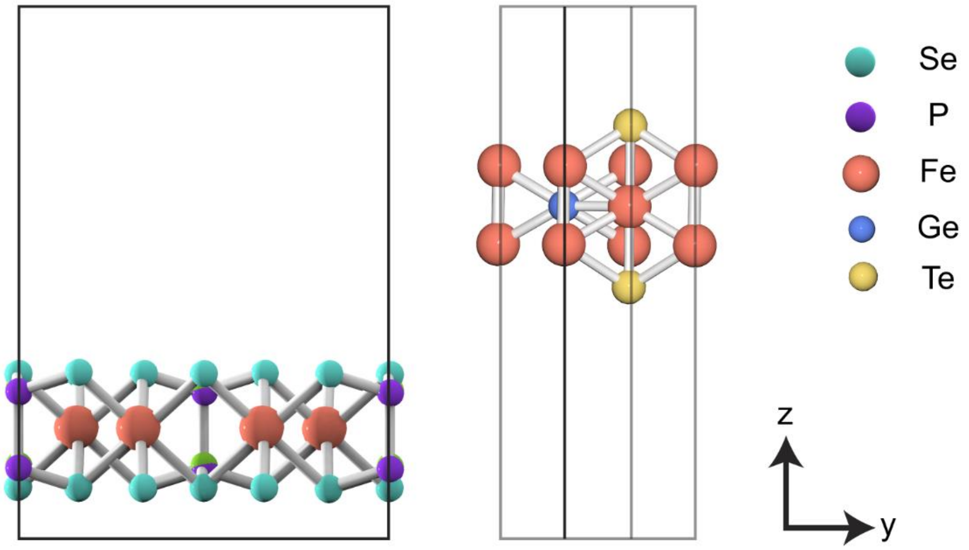

Supplementary Fig. 5 | Unit cell of FPSe and FGT. The unit cell FPSe (left) and FGT (right) built using BURAI (Quantum Espresso GUI).

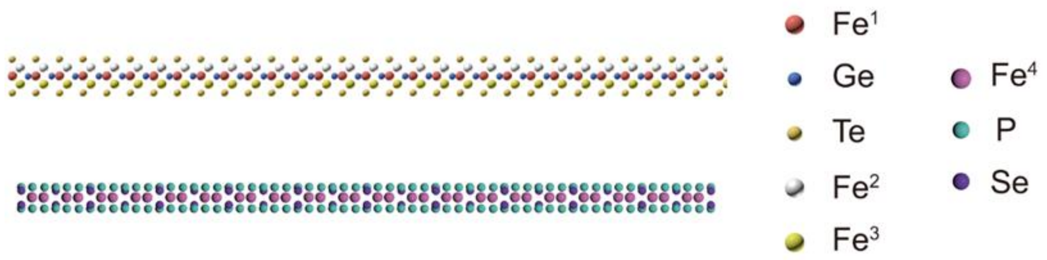

Supplementary Fig. 6 | Single-layer structures of FPSe and FGT. Layers in Molecular Dynamics simulations using LAMMPS for FPSe (bottom) and for FGT (top). On the FGT flake, the top, and bottom light-yellow atoms are Te atoms, the following white, darker yellow and red atoms are Fe atoms, and the blue atoms are Ge atoms. On the FPSe flake, the light blue atoms are Se atoms, the purple atoms are P atoms, and the pink atoms are Fe atoms.

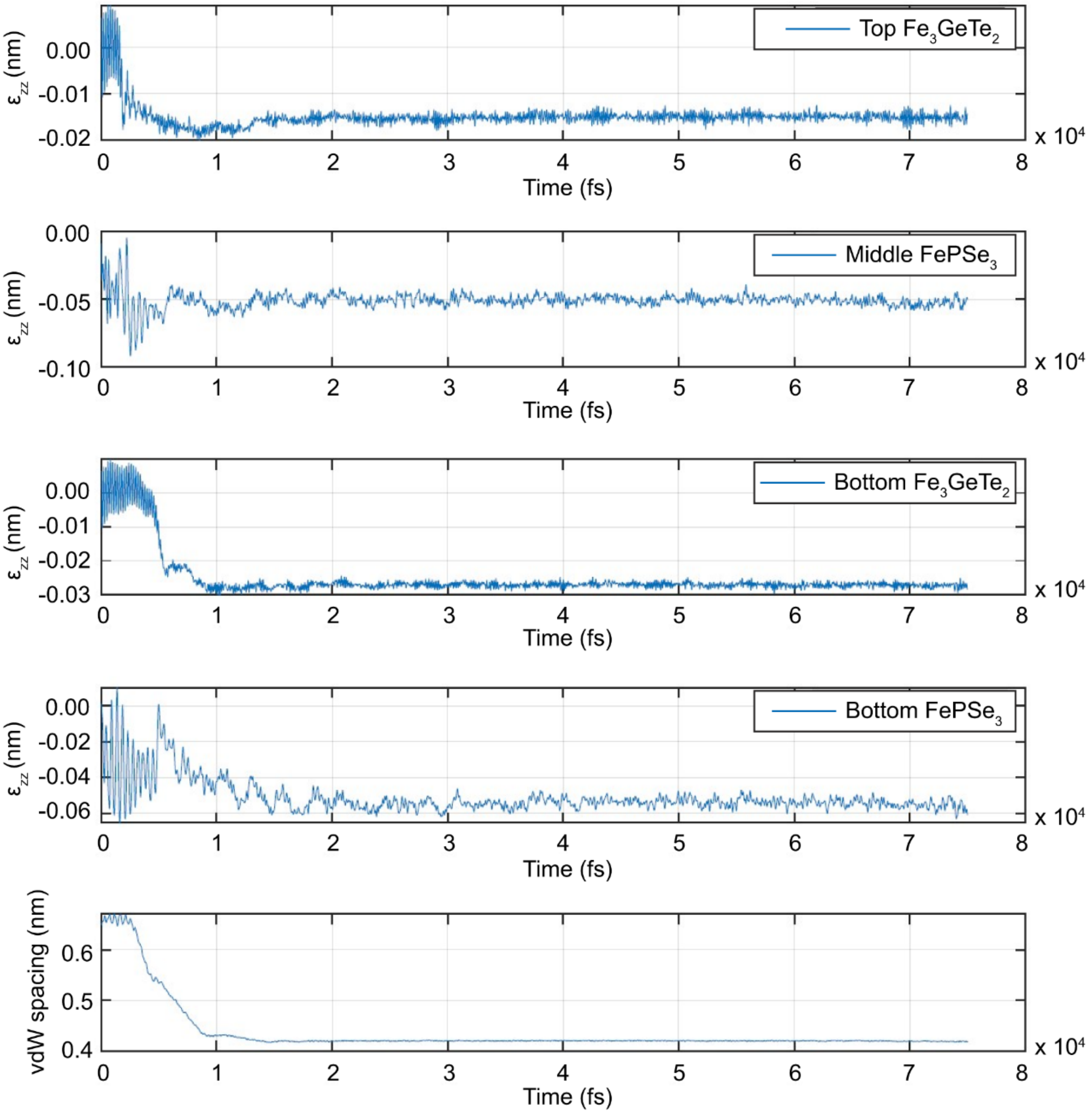

Supplementary Fig. 7 | Strain and interlayer distance evolution in the laser shock MD simulation. Strain evolution in the laser shock MD simulation is shown for top FGT, middle FPSe, bottom FGT, and bottom FPSe, respectively. The interlayer distance of heterostructure is reduced from 0.66 nm to 0.42 nm.

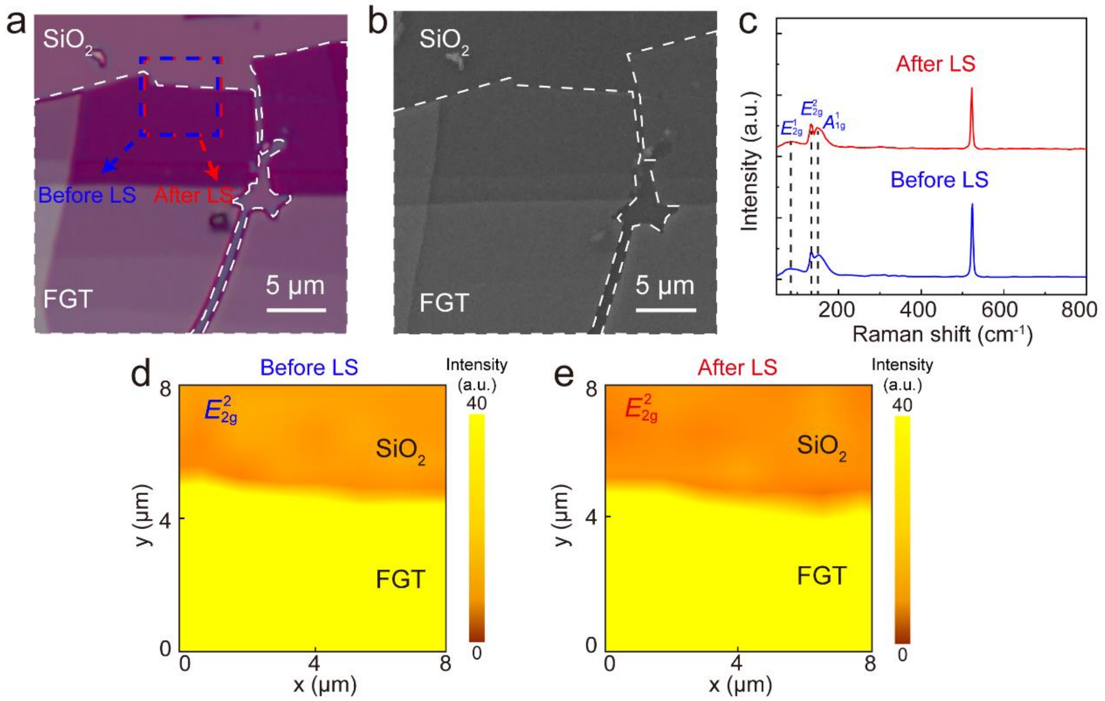

Supplementary Fig. 8 | Characterization of FGT after LS. a, b The optical image and the SEM image of FGT after LS. **c** Raman spectrum of FGT before and after LS. **d, e** Raman mapping of E_{2g}^{2} peak intensity of FGT flake before (**d**) and after (**e**) LS. The corresponding scanning areas before and after LS were marked by the blue and red boxes in (a) respectively.

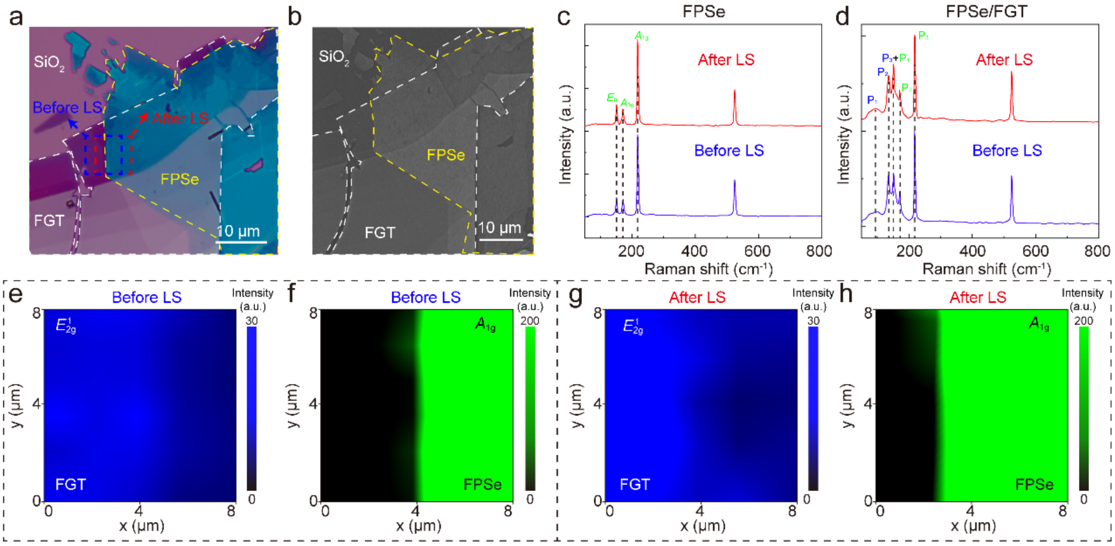

Supplementary Fig. 9 | Characterization of FPSe and FPSe/FGT heterostructure after LS. a, b The optical image and the SEM image of FPSe/FGT heterostructure after LS. **c, d** Raman spectrum of FPSe and FPSe/FGT heterostructure before and after LS. In Fig. 2d, P_1, P_2, and P_3 marked in green in (d) represent the characteristic peak of FPSe flake in (c). P_1, P_2, and P_3 marked in blue represent the characteristic peaks E^1_{2g}, E^2_{2g}, and A^1_{1g} of FGT flake. **e-h** Raman mapping of FPSe/FGT heterostructure before (**e, f**) and after (**g, h**) LS. The blue mappings represent E^1_{2g} peak (~90 cm^{-1}) intensity of FGT flake and the green mappings represent A_{1g} peak (~215 cm^{-1}) intensity of FPSe flake. The areas marked by the blue and red boxes in (a) represent the test ranges before and after LS respectively.

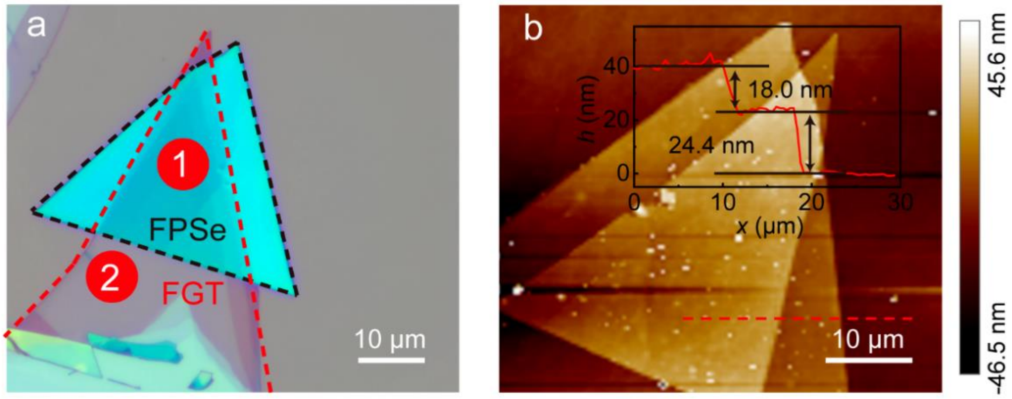

Supplementary Fig. 10 | Characterization of sample A. a, b The optical microscope image (**a**) and the atomic force microscope image (**b**) of sample A. The thickness of FPSe and FGT is 18.0 nm and 24.4 nm, respectively.

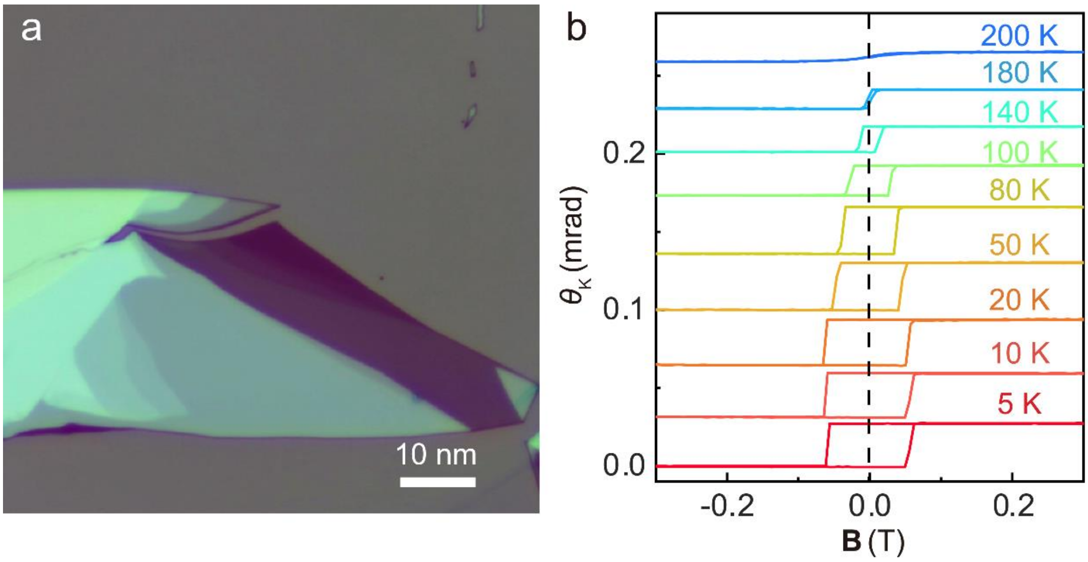

Supplementary Fig. 11 | The Kerr hysteresis loops of pure FGT. a, The optical microscope image of pure FGT. **b**, The temperature-dependent Kerr loops for pure FGT, showing a lack of H_{EB}.

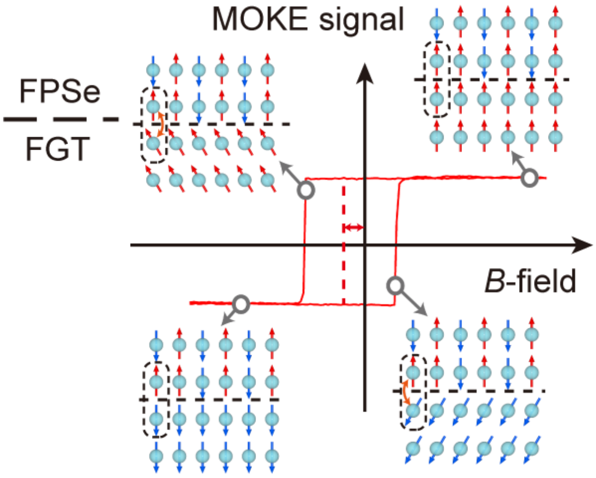

Supplementary Fig. 12 | The schematic diagram of the M-B model.

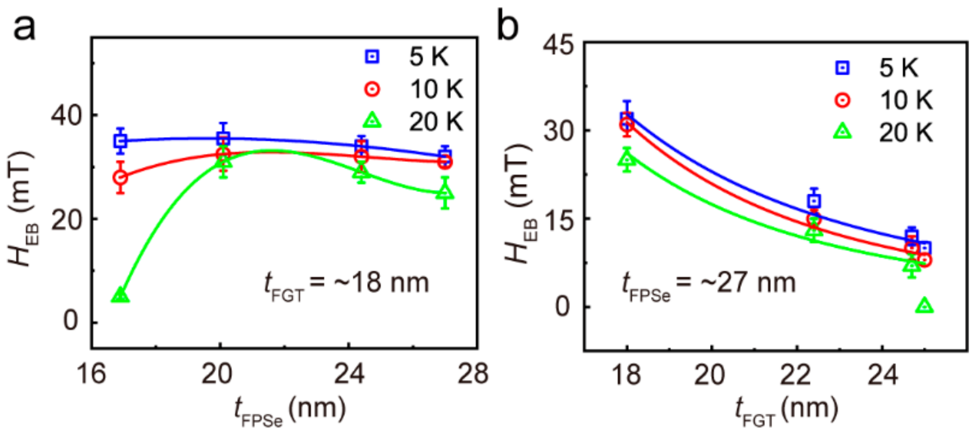

Supplementary Fig. 13 | The thickness dependences of H_{EB} in AFM/FM systems. **a**, **b** the t_{FPSe} dependences (**a**) and the t_{FGT} dependences (**b**) of H_{EB} in our exchange-biased AFM/FM systems. Error bars represent standard deviation for several consecutive measurements.

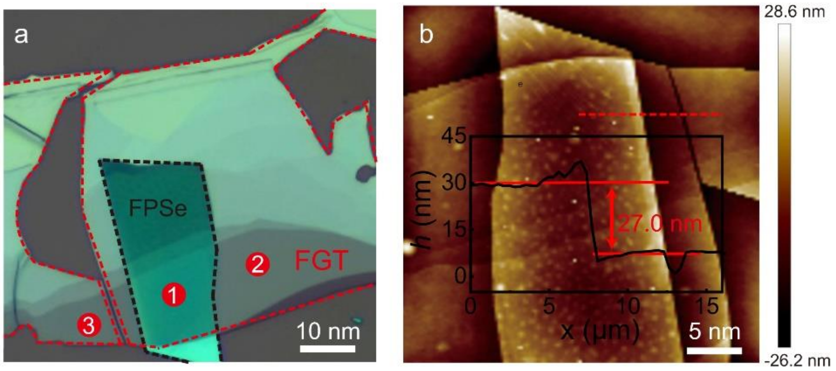

Supplementary Fig. 14 | Characterization of sample B. a, b The optical microscope image (**a**) and the atomic force microscope image (**b**) of sample B. Test positions 1, 2 and 3 represent FPSe/FGT, connected FGT, and isolated FGT, respectively.

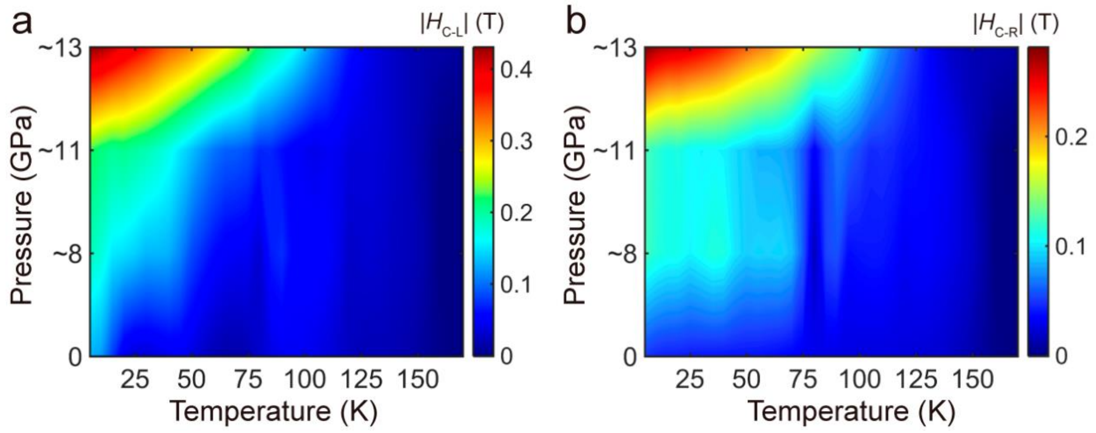

Supplementary Fig. 15 | The evolution of the coercive field on temperature and pressure. a, b The evolution of $H_{C\text{-}L}$ and $H_{C\text{-}R}$ on temperature and pressure before and after LS.

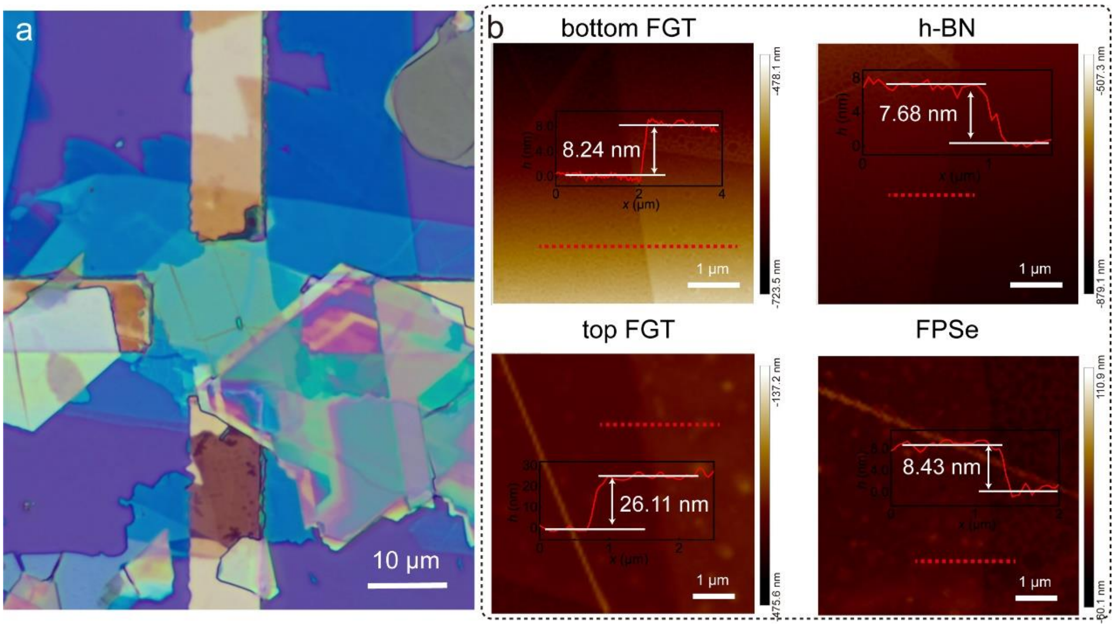

Supplementary Fig. 16 | Characterization of FPSe/FGT/h-BN/FGT heterostructure. The optical microscope image and the atomic force microscope image of FPSe/FGT/h-BN/FGT heterostructure (pristine device and LS modulation device).

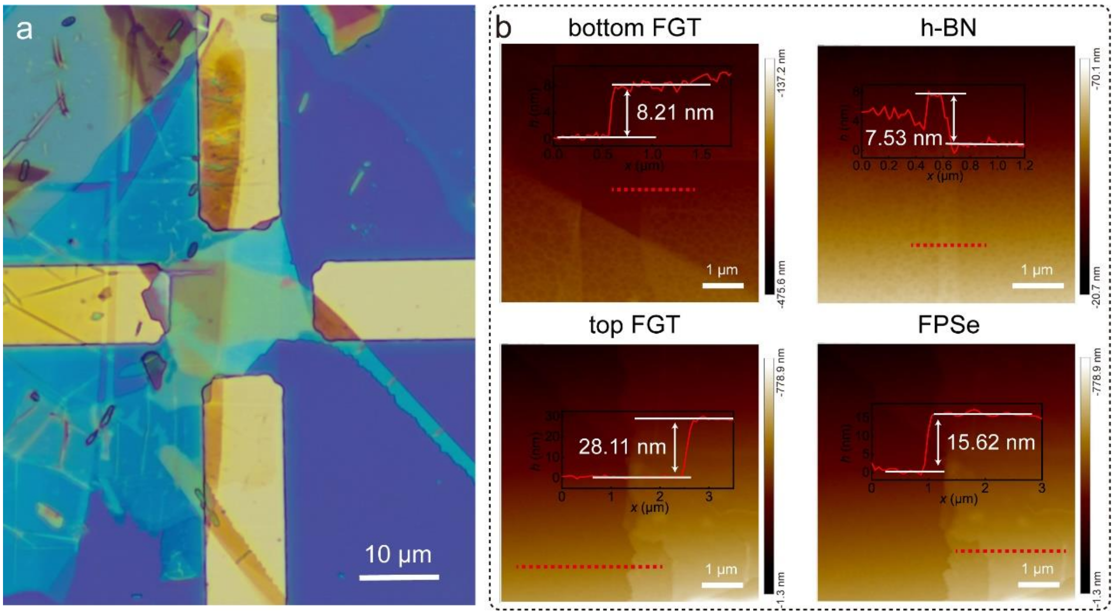

Supplementary Fig. 17 | Characterization of FPSe/FGT/h-BN/FGT heterostructure. The optical microscope image and the atomic force microscope image of FPSe/FGT/h-BN/FGT heterostructure (thickness modulation device).

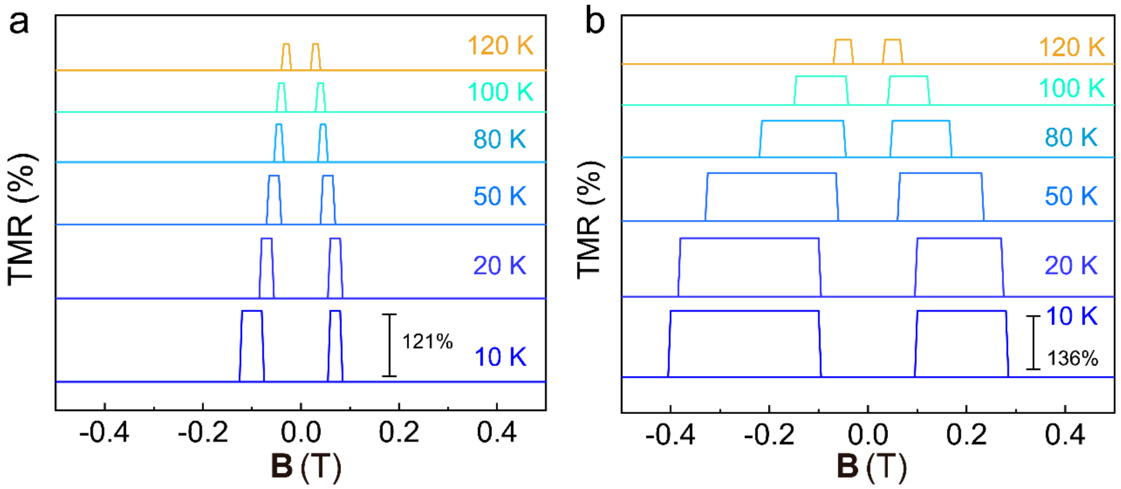

Supplementary Fig. 18 | TMR measurement of a FPSe/FGT/h-BN/FGT vdW heterostructure. TMR curves measurement of FPSe/FGT/h-BN/FGT at different temperatures before and after LS. The bias current is fixed at 10 μA.

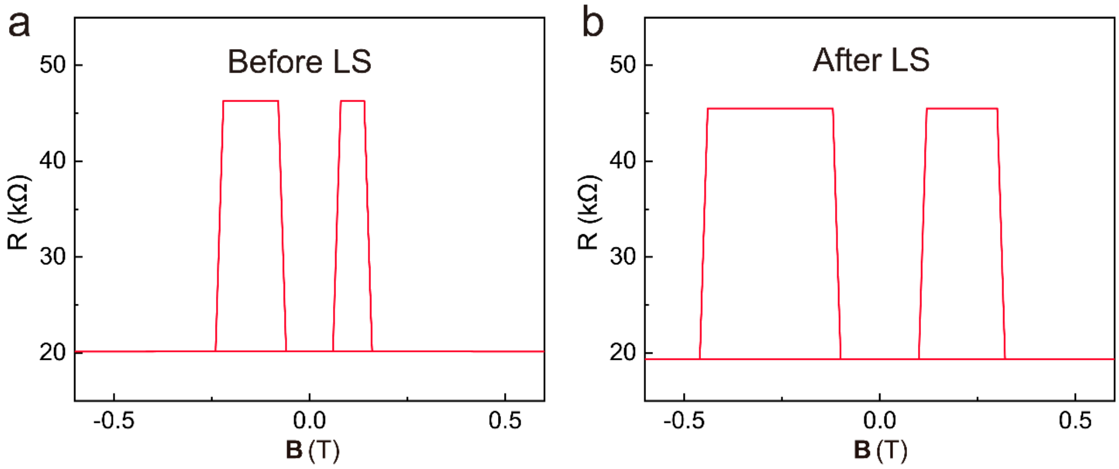

Supplementary Fig. 19 | TMR measurement of thickness modulation device. The tunnel resistance of thickness modulation device before and after LS. The bias current is fixed at 10 μA.

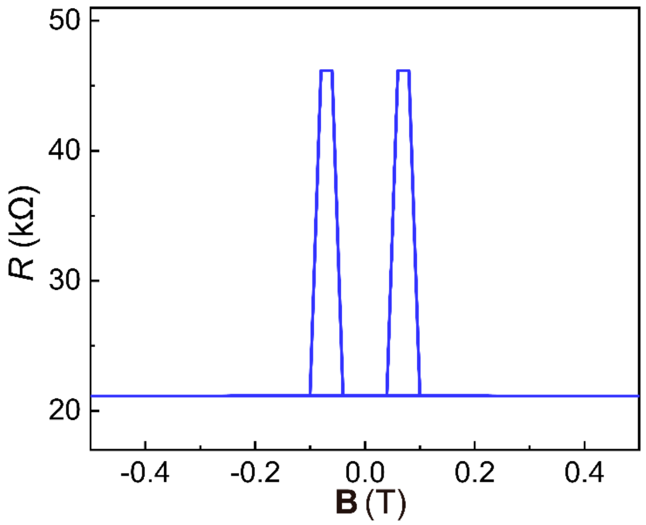

Supplementary Fig. 20 | TMR measurement of FGT/h-BN/FGT vdW heterostructure. TMR curve was measured at 5 K. The bias current is fixed at 10 μA.

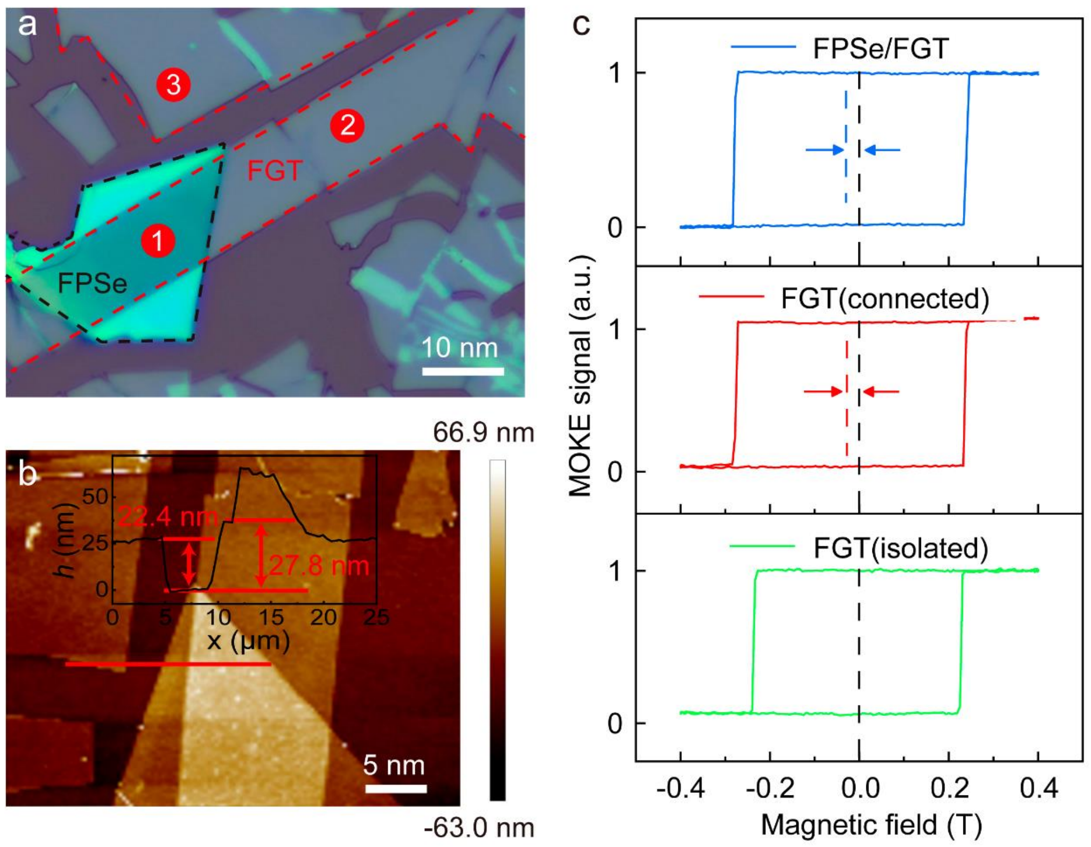

Supplementary Fig. 21 | Characterization and MOKE signal for sample C. a, b The optical image and the atomic force microscope image sample C. Position 1, 2, and 3 indicate FPSe/FGT, connected FGT, and isolated FGT, respectively. **c** MOKE signal for isolated FGT, connected FGT, and FPSe/FGT heterostructures, respectively, measured at 5 K. When FGT is connected to FPSe/FGT heterostructures, a consistent exchange bias effect occurs. In contrast, FGT is not affected by the magnetic properties of the heterostructure.

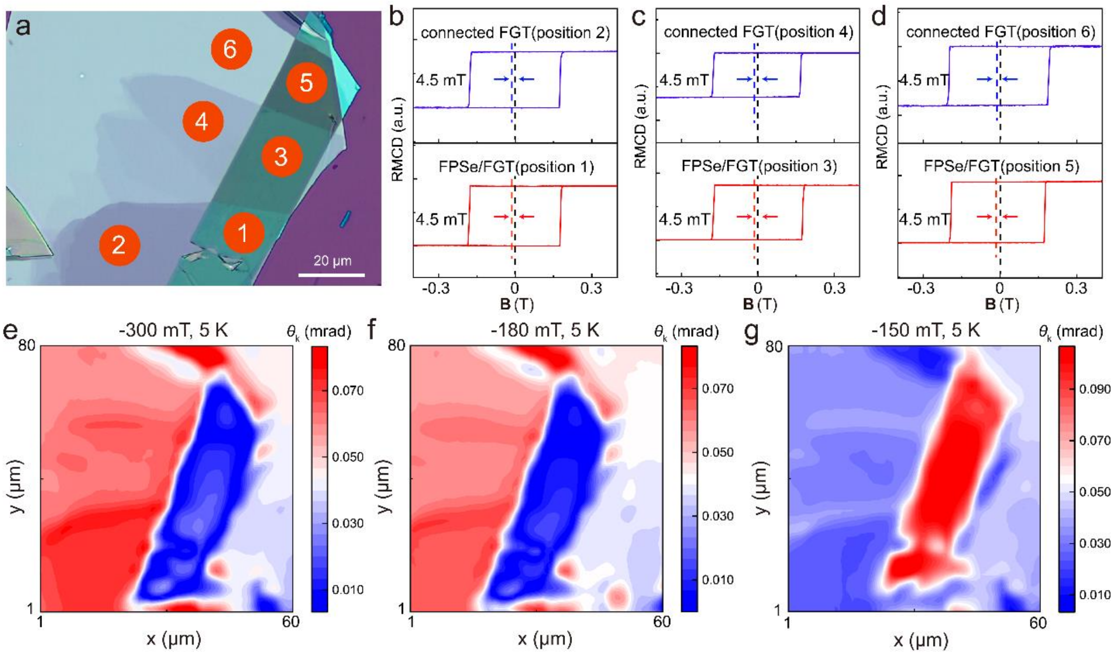

Supplementary Fig. 22 | RMCD signal of FPSe/FGT heterostructure (sample D). **a** The optical image of FPSe/FGT heterostructure. Positions 1,3, and 5 represent the heterostructure regions, and positions 2,4, and 6 represent the adjacent connected bare FGT regions. **b-d** the RMCD signals of positions 1 and 2 (**b**), 3 and 4 (**c**), and 5 and 6 (**d**), respectively. **e-g** MOKE mappings under the different magnetic fields for the FPSe/FGT heterostructure. All the data was measured at 5 K.

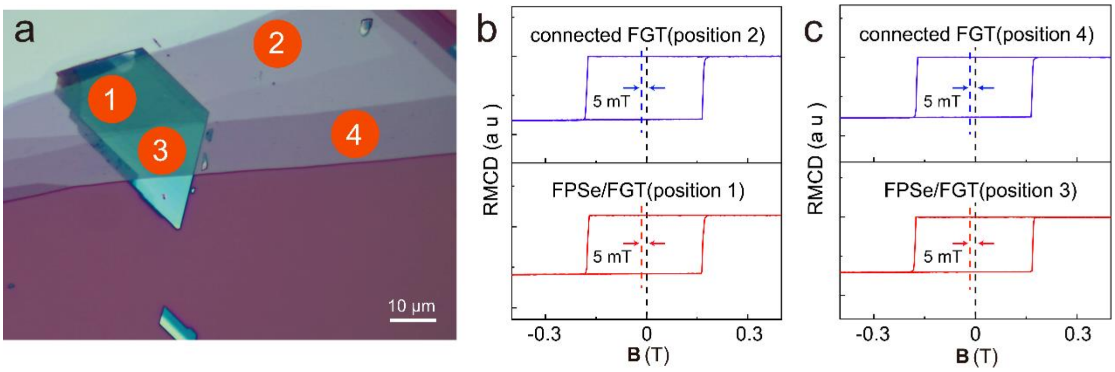

Supplementary Fig. 23 | RMCD signal of FPSe/FGT heterostructure (sample E). a The optical image of the FPSe/FGT heterostructure. Positions 1 and 3 represent heterostructure regions, and positions 2 and 4 represent the adjacent connected bare FGT regions. **b, c** the RMCD signals of positions 1 and 2 (**b**), 3 and 4 (**c**), respectively.

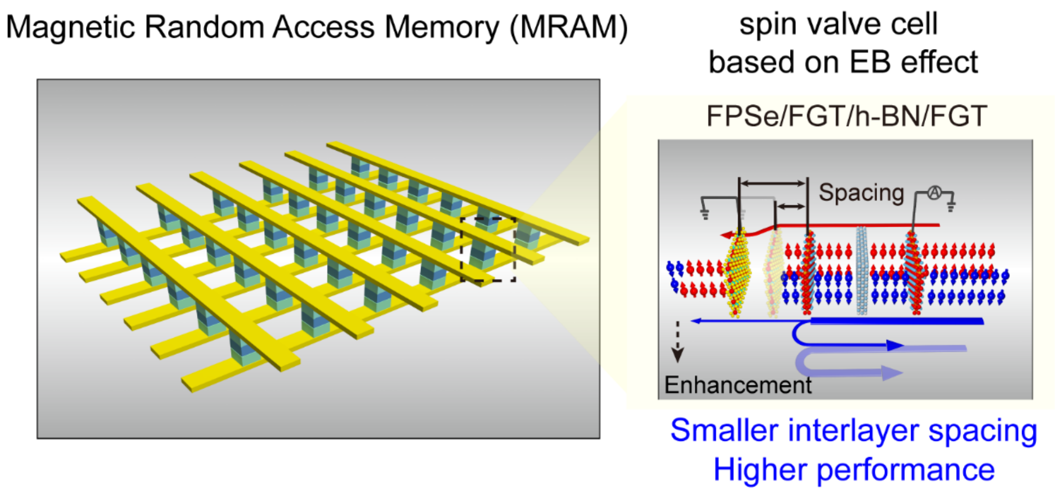

Supplementary Fig. 24 | Schematic diagram of Magnetic Random Access Memory (MRAM). Unit cell of MRAM is a spin valve device based on EB effect, whose performance can be improved by reducing the layer spacing.

Table 1. Atomic positions (Å) in simulation cells at the ground state (image from BURAI).

Flake	Atom	X coordinate	Y coordinate	Z coordinate
$FePSe_3$	P	5.529822	0	1.470897
	P	-0.63637	0	3.533669
	P	2.446727	5.340123	1.427536
	P	2.446727	5.340077	3.533669
	Fe	-0.63637	7.120149	2.520469
	Fe	5.529822	3.560051	2.440735
	Fe	2.446727	1.780026	2.520469
	Fe	2.446727	8.900174	2.440735
	Se	4.767796	5.371055	1.097007
	Se	4.3425	1.994639	1.097007
	Se	4.396075	8.65463	1.097007
	Se	1.684701	0.030932	1.097007
	Se	1.31298	3.314507	1.097007
	Se	1.259405	7.334762	1.097007
	Se	3.634049	3.345438	3.864199
	Se	3.580474	7.365693	3.864199
	Se	0.550954	8.685561	3.864199
	Se	0.497379	2.02557	3.864199
	Se	3.208753	10.64927	3.864199
	Se	0.125658	5.309145	3.864199
Fe_3GeTe_2	Fe	1.9955	1.152102	10
	Te	1.9955	1.152102	12.61
	Te	1.9955	1.152102	7.39
	Fe	0	0	11.28
	Fe	0	0	8.72
	Ge	0	2.304205	10

Table 2. Energy states for different atomic configurations.

Configuration	System Energy (eV)	Energy difference compared to the Ground-state (eV)
Ground state	-2881.9	0
State 1	-2881.86	0.042627
State 2	-2881.89	0.006938
State 3	-2881.85	0.049447

Table 3. Interatomic interactions model parameters. The superscripts for Fe atoms denote their locations as shown in Figure S9.

Interaction type	Interaction model	Atoms involved	ε (eV)	σ (Å)	Z (eV)
Fe_3GeTe_2 (interlayer)	Axilrod Teller	Fe^1-Fe^1-Fe^1	---	---	10
	Axilrod Teller	Fe^1-Fe^1-Ge	---	---	10
	Axilrod Teller	Fe^1-Fe^1-Te	---	---	10
	Lenard Jones	Fe^1-Fe^1	0.1	3.555577	---
	Lenard Jones	Fe^1-Ge	0.1	2.052813	---
	Lenard Jones	Fe^1-Te	0.9	2.325246	---
	Lenard Jones	Fe^1-Fe^2	0.15	2.348285	---
	Lenard Jones	Fe^1-Fe^3	0.15	2.348285	---
	Lenard Jones	Ge-Ge	0.15	3.555577	---
	Lenard Jones	Ge-Te	0.15	3.101743	---
	Lenard Jones	Ge-Fe^2	0.15	2.348285	---
	Lenard Jones	Ge-Fe^3	0.15	2.348285	---
	Lenard Jones	Te-Te	0.1	3.555577	---
	Lenard Jones	Te-Fe^2	0.15	2.370236	---
	Lenard Jones	Te-Fe^3	0.15	2.370236	---
	Lenard Jones	Fe^2-Fe^2	0.1	3.555577	---
	Lenard Jones	Fe^2-Fe^3	0.9	2.280701	---
	Lenard Jones	Fe^3-Fe^3	0.1	3.555577	---
$FePSe_3$ (interlayer)	Axilrod Teller	Fe^4-Fe^4-Se	---	---	101
	Axilrod Teller	Fe^4-P-Se	---	---	101
	Axilrod Teller	P-P-Se	---	---	101
	Axilrod Teller	Fe^4-Se-Se	---	---	101
	Axilrod Teller	P-Se-Se	---	---	101
	Lenard Jones	Fe^4-Fe^4	0.01	3.17249	---
	Lenard Jones	Fe^4-P	0.4	3.305345	---
	Lenard Jones	Fe^4-Se	0.22	2.144372	---
	Lenard Jones	P-P	0.04	1.934383	---
	Lenard Jones	P-Se	0.18	2.090184	---
	Lenard Jones	Se-Se	0.04	3.276599	---
Fe_3GeTe_2/$FePSe_3$ (interlayer)	Lenard Jones	Te-P	0.01	3.85	---
	Lenard Jones	Te-Se	0.001	6.4	---
	Lenard Jones	Fe^2-P	0.6	4.9	---
	Lenard Jones	Fe^2-Se	0.85	4.65	---
	Lenard Jones	Fe^3-P	0.6	4.9	---
	Lenard Jones	Fe^3-Se	0.85	4.65	---

Table 4. The peak pressures at 4 different laser intensities.

Average power (W)	Peak power density (GW cm^{-2})	Peak pressure (GPa)
2	2.0376	5.31
5	5.094	8.41
9	9.1692	11.26
13	13.2444	13.54

Table 5. The performance comparison before and after laser shocking

	before laser shocking	after laser shocking
Coercive field (left)	0.126 T	0.426 T
Coercive field (right)	0.061 T	0.277 T
Exchange bias field	32 mT	74 mT
Blocking temperature	20 K	110 K

References

1 Borysiuk, V. N., Mochalin, V. N. & Gogotsi, Y. Molecular dynamic study of the mechanical properties of two-dimensional titanium carbides Ti(n+1)C(n) (MXenes). *Nanotechnology* **26**, 265705 (2015).

2 Motlag, M. *et al*. Laser-Shock-Induced Nanoscale Kink-Bands in WSe_2 2D Crystals. *ACS Nano* **13**, 10587-10595 (2019).

3 Gao, H. *et al*. Nanolithography. Large-scale nanoshaping of ultrasmooth 3D crystalline metallic structures. *Science* **346**, 1352-1356 (2014).

4 Fabbro, R., Fournier, J., Ballard, P., Devaux, D. & Virmont, J. Physical study of laser-produced plasma in confined geometry. *J. Appl. Phys.* **68**, 775-784 (1990).

5 Rathod, V. T. A Review of Acoustic Impedance Matching Techniques for Piezoelectric Sensors and Transducers. *Sensors (Basel)* **20,** 4051 (2020).

6 Zheng, Q., Braun, P. V. & Cahill, D. G. Thermal Conductivity of Graphite Thin Films Grown by Low Temperature Chemical Vapor Deposition on Ni (111). *Advanced Materials Interfaces* **3,** 1600234 (2016).
